# A Classic Herbal Formula Danggui Beimu Kushen Wan for Chronic Prostatitis: From Traditional Knowledge to Scientific Exploration

**DOI:** 10.1155/2018/1612948

**Published:** 2018-11-13

**Authors:** Hong Li, Andrew Hung, Angela Wei Hong Yang

**Affiliations:** ^1^School of Health and Biomedical Sciences, RMIT University, Australia; ^2^School of Science, RMIT University, Australia

## Abstract

Chronic prostatitis (CP) is a chronic inflammation in the prostate with unsatisfactory management. Danggui Beimu Kushen Wan (DBKW) is a classic formula developed 1800 years ago for patients with difficult urination and it has been widely utilized for CP in modern days. However, scientific understanding of DBKW on CP has not been systematically reviewed. First, we searched the Encyclopedia of Traditional Chinese Medicine for the etiologies and pathogeneses of CP-like symptoms and DBKW and compared their similarities and differences from traditional Chinese medicine and conventional medicine perspectives. Then, we searched 21 electronic databases to identify potential clinical and experimental studies. Characteristics of included studies, ingredients, herb frequency, and possible mechanisms of actions were descriptively summarized. Risk of bias of randomized controlled trials (RCTs) was evaluated using the Cochrane risk of bias assessment tool. A total of 290 studies were identified. Six clinical studies, including four RCTs and two case series, and eight experimental studies were included. Clinical studies indicated that DBKW used alone or as an adjunct therapy significantly reduced the CP symptom scores and decreased the expressed prostatic secretions-pH when compared to antibiotics or *α*-blocker. Most RCTs have high or unclear risk of bias. Experimental studies revealed that DBKW may have effects on anti-inflammation, antibacteria, antioxidation, sex hormone regulation, and immunoregulation. DBKW demonstrated a great potential in the treatment of CP. High-quality RCTs and network pharmacological studies should be considered for future research.

## 1. Introduction

Prostatitis is a common inflammatory prostate disorder that affects approximately 30% to 50% of men [1, 2]. A systematic review involving more than 10,000 patients claimed that the prevalence of prostatitis symptoms is around 8.2% [1]. Men aged 36 to 50 years seem to be prone to have this condition although it impacts men of all ages [3]. The National Institute of Health of United States (NIH) defines the chronic types of prostatitis as chronic bacterial prostatitis (CBP) and chronic prostatitis/chronic pelvic pain syndrome (CP/CPPS) [4]. Urogenital pain, sexual dysfunction, psychological symptoms, and lower urinary tract symptoms (i.e., frequent, urgent, and painful micturition) are the primary chronic prostatitis-related symptoms [5]. Conventional management for CBP and CP/CPPS includes medications (i.e., antibiotics, *α*-blocker, anti-inflammatory, and 5*α*-reductase-inhibitors), surgery, physiotherapies, and psychotherapies [6, 7]. Recurrences are common when patients stop using medications [8]. A study which enrolled 68,675 patients pointed out that men who suffered from CP had a 30% higher probability of developing prostate cancer (PCa) than others [9]. However, the treatment effects of current management are unsatisfactory.

There is a trend to identify potential treatment from traditional knowledge to guide modern drug discovery [10]. In antiquity, terminologies for prostate or prostate disorders (such as CP, CBP, and CPPS) did not exist. However, relevant records can be found to describe prostatitis-related symptoms such as Jing Zhuo in traditional Chinese medicine (TCM) [11], with applications of various herbal formulas [12–14]. Within all records of formulas, Danggui Beimu Kushen Wan (Chinese Angelica, Fritillaria and Flavescent Sophora Pill; DBKW) is one of the formulas that are indicated for difficult urination in pregnant women or male patients. In the modern era, this formula has been widely utilized for a range of prostate disorders involving CP [15], benign prostatic hyperplasia [16], and PCa [17]. This formula containing* Angelicae Sinensis Radix* (Dang gui),* Fritillariae Thunbergii Bulbus* (Zhe bei mu),* Sophorae Flavescentis Radix* (Ku shen), and* Talcum* (Hua shi) was initially created by ZHANG Zhong-jing (150-219 AD), the Chinese medical sage, detailed in Jin Gui Yao Lue (Synopsis of Prescriptions of the Golden Chamber; JGYL), approximately 1800 years ago [18]. However, its applications have not been systematically reviewed. This study aimed to systematically review the existing clinical and experimental studies of DBKW.

## 2. Pathophysiology of CP in TCM and Conventional Medicine

**Figure 1:**
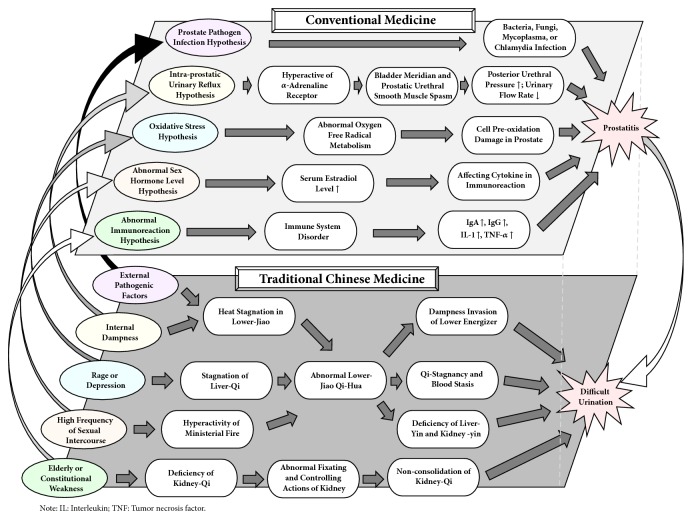
Etiologies and pathogeneses of chronic prostatitis in traditional Chinese medicine and conventional medicine and their relationship.

We searched Zhong Hua Yi Dian (Encyclopedia of Traditional Chinese Medicine; ZHYD), a CD-ROM containing 1,156 ancient books, to identify potential classic literature of Jing Zhuo and DBKW [11]. The original record of “Jing Zhuo” in TCM could be traced back to Mi Chuan Zheng Zhi Yao Jue (Essentials for Esoteric Treatment; DAI Yuan-li; 1443) and it was caused by stagnation in water passage. Then a series of classic literature also documented its etiologies and pathogeneses in different aspects. Ben Cao Pin Hui Jing Yao (Collected Essentials of Species of Materia Medica; LIU Wen-tai; 1505) described the systems of Jing Zhuo mainly presented in difficult urination with white discharge in urinary meatus, due to hyperactivity of fire or yin deficiency. Wang Xu Gao Lin Zheng Yi An (Wang Xugao's Clinical Cases; WANG Xu-gao; 1897) believed that it resulted from external dampness and heat while Mei Shou Tang Fang An Xuan Cun (Mei Shou Tang's Selected Cases; YE Tian-shi; n.d) thought internal dampness and heat led to Jing Zhuo. In addition, Zang deficiency described in Yi Xue Ru Men (Introduction to Medicine; LI Ting; 1575), high frequency of sexual intercourse documented in Fu Xi Mi Chuan Jian Yan Fang (Fu Xi's Esoteric Experienced Formulae; LU Jin-sui; 1918), and Liver-qi stagnation recorded in Mai Jian Bu Yi (Annotation of Pulse Diagnosis; ZHOU Xue-hai; 1892) were the other three reasons that caused Jing Zhuo.  Figure 1 shows the common etiologies and pathogeneses of Jing Zhuo in TCM based on the description in the textbook of Surgery of Chinese Medicine [19]. On the other hand, although the exact pathophysiology of CP is unknown, five primary hypotheses were proposed in modern research, including prostate pathogen infection [20, 21], intraprostatic urinary reflux [22–24], oxidative stress [25–27], abnormal sex hormone level [28–31], and abnormal immunoreaction [27, 32–34]. Detailed pathophysiology of prostatitis in conventional medicine and the relationship between TCM and conventional medicine are illustrated in Figure 1.

Compared to the etiologies and pathogeneses of Jing Zhuo recorded in classic literature, it is not difficult to find that there are some similarities in TCM and conventional medicine. TCM believes that all pathogens causing infections, such as viruses, bacteria, fungi, mycoplasma, and chlamydia belong to the category of external pathogenic factors, whereas internal dampness may be produced when the urinary reflux exists, according to the intraprostatic urinary reflux hypothesis [35]. Recent research has shown that oxidative stress may be relevant to emotional stress (i.e., excessive rage or depression) [36, 37] or irregular lifestyle [38]. Moreover, the frequency of sexual intercourse and sex hormone level may have a certain connection with each other [39] that could also be considered to belong to the category of irregular lifestyle in TCM or conventional theories, causing the development of CP [40]. Patients who are elderly or constitutionally weak may be more prone to abnormal immunoreaction that may lead to CP [41]. Based on the above findings, both TCM and conventional medicine have their own understanding of prostatitis. However, it is essential to be aware that these etiologies and pathogeneses arrive at the same end by different means.

In JGYL, DBKW was documented to treat difficult urination [18]. Some classic TCM literature recorded its pathogenesis [11]. For example, Chang Sha Yao Jie (Changsha Explanation of Medicines; HUANG Yuan-yu; 1753) and Jin Gui Xuan Jie (Explanation to the Synopsis of Golden Chamber; HUANG Yuan-yu; 1754) recorded that abnormal bladder Qi-Hua was one of the pathogeneses that cause difficult urination. The other one was heat stagnation in Lower-Jiao, which could be detected in Jin Gui Yao Lue Guang Zhu (Extensive Commentary to the Treatise on the Synopsis of Prescriptions of the Golden Chamber; LI Wen; 1682), Tai Chan Xin Fa (Experience in Obstetrics; Yan Chun-xi; 1730), and Nu Ke Yao Zhi (Essential Guide of Gynecology, CHEN Nian-zu; 1804). Moreover, Lin Zheng Zhi Nan Yi An (Case Records as a Guide to Clinical Practice; LZZNYA; YE Tian-shi; 1746) distinctly pointed out that the primary rule of treatment for Jing Zhuo was clearing dampness and heat. Compared to the description in LZZNYA as well as the pathogeneses of CP listed in Figure 1, these two pathogeneses of DBKW partially overlap and match the pathogeneses of prostatitis.

## 3. Modern Scientific Exploration of DBKW on CP

We searched the following 21 electronic bibliographic English and Chinese databases from their respective inception to 17 April 2018 to identify potential clinical and experimental studies, including the Cochrane Central Register of Controlled Trials, PubMed, EMBASE, CINAHL, Informit, Science Direct, LILACS (Latin American and Caribbean Health Sciences), ProQuest, AMED, Blackwell Synergy, PsycINFO, INDMED, AcuBriefs, Ingenta, Koreamed, ERIC, mRCT, CNKI, CQVIP, Wanfangdata, and CBM. Keywords used to identify clinical studies included Danggui Beimu Kushen Wan, clinical studies, clinical trials, case reports, and their synonyms. Keywords used to identify experimental studies included Danggui Beimu Kushen Wan, experimental studies,* in vivo*,* in vitro*, and their synonyms. A manual search was performed to screen the reference list of review articles for potential studies.

For clinical studies, all types of clinical studies (e.g., RCTs and case series), published in English or Chinese, were considered, regardless of the type of publication. The studies were included if they focused on CP using DBKW or modified DBKW as the intervention. For controlled studies, the control group can be a conventional medicine, placebo or no treatment. The literature was excluded if the participants were not suffering from CP, or the formula used in the study was not modified from DBKW, or diagnosis of CP was not provided, or an inappropriate control group was involved. The experimental studies (e.g., animal studies,* in vivo*,* in vitro*) related to the mechanisms of action of DBKW for the management of CP were included in the review. The literature was excluded if the studies were not for CP.

One reviewer (HL) screened the included clinical and experimental studies and extracted the following data to two predesigned Excel spreadsheet templates: (1) study type, sample size, dropouts, diagnosis, interventions, duration, outcome measurement, adverse events, follow-up, and herb ingredients for clinical studies; (2) animal types, sample size, diagnosis, interventions, duration, outcome measurement, and herb ingredients for experimental studies. Risk of bias of the included RCTs was assessed by one reviewer (HL) with the Cochrane risk of bias assessment tool [42] as well. The second reviewer (AY) checked and confirmed the data and assessment process. Any discrepancies between the two reviewers were resolved through discussion with the third party (AH). Characteristics of included clinical and experimental studies, herb ingredients, herb frequency, study results, and possible pharmaceutical effects were descriptively summarized.

**Figure 2:**
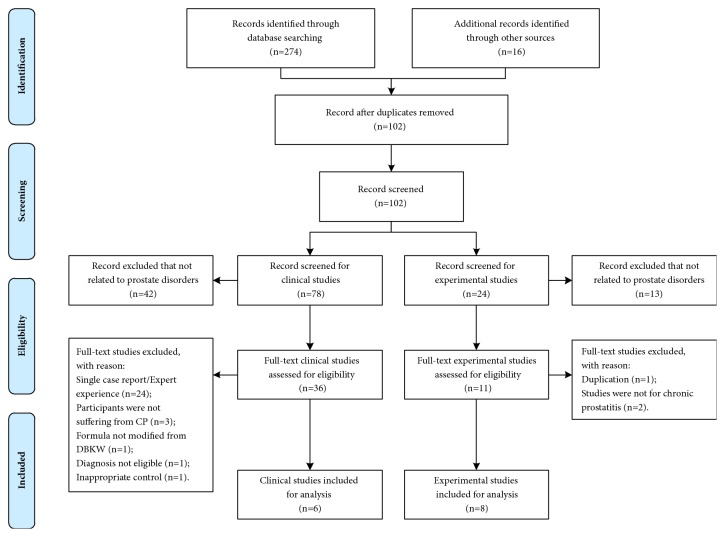
Flowchart of the selection process for identifying studies to be included in the review.

A total of 290 clinical and experimental studies were identified following the search strategies outlined above. Six clinical studies and eight experimental studies meeting the inclusion criteria were included in the review.  Figure 2 illustrates the detailed selection process.

### 3.1. Overall Clinical Implications of DBKW on CP

Six clinical studies were included in the review, involving four RCTs [12, 15, 43, 44] and two case series [45, 46]. Characteristics of six identified clinical studies were summarized in Table 1.

Among six included studies, one RCT focused on the treatment of CBP [15], one RCT [12] and one case series [45] for CP/CPPS, two studies for both CBP and CP/CPPS (one RCT [43] and one case series [46]), and one RCT for CP without any specified category [44]. In terms of herbal intervention, five studies applied modified DBKW decoction as an intervention [15, 43–46] and the other one utilized original DBKW decoction [12]. Three RCTs compared modified DBKW with western drugs [15, 43, 44] and one RCT compared original DBKW plus *α*-blocker with the same *α*-blocker only [12]. Details of herb ingredients in the included clinical studies are presented in Table 2.

There are a total 19 herbs used in basic prescriptions in the included studies and eight herbs were utilized more than once, including* Angelicae Sinensis Radix* (Dang gui; six times),* Fritillariae Thunbergii Bulbus* (Zhe bei mu; six times),* Sophorae Flavescentis Radix* (Ku shen; six times),* Talcum* (Hua shi; five times),* Vaccariae Semen* (Wang bu liu xing; twice),* Phellodendri Chinensis Cortex* (Huang bai; twice),* Taraxaci Herba* (Pu gong ying; twice), and* Cyathulae Radix* (Chuan niu xi; twice). These herbs are from heat-clearing, blood-invigorating and blood stasis-removing, and tonifying categories. Due to the significant variety of herbs used in the included studies, it is infeasible to assess the effects of the basic formula (i.e., DBKW) across different studies. It is recommended that future research considers using the formula with original ingredients to ensure the possibility of data synthesis.

None of the included RCTs involved placebo or any treatment in the control group. All of them utilized conventional medicines (antibiotic or *α*-blocker drugs) as the comparators [12, 15, 43, 44]. Both Guo [43] and Wu [15] claimed that the total effectiveness rate of modified DBKW was superior to antibiotic drugs; however, detailed data are not available for further synthesis. Another RCT comparing DBKW with ofloxacin indicated that DBKW could significantly decrease pH level in the expressed prostatic secretions test (EPS) (mean difference (MD) -0.17, 95% confidence interval (CI) -0.33 to -0.01) [44]. Although Wu [44] claimed that statistical significance existed in the improvement rate of EPS-white blood cell counts between DBKW and ofloxacin, the effect size analysis did not support this finding (odds ratio (OR) 1.72, 95% CI 0.63 to 4.72) and neither did the EPS-lecithin body between DBKW and ofloxacin (MD -0.02, 95% CI -0.32 to 0.28). Furthermore, the trial from Zhang, Yang, and Chen revealed that the adjunct effects of basic DBKW decoction were superior to terazosin hydrochloride, evidenced by reducing the score of NIH-Chronic Prostatitis Symptom Index (NIH-CPSI) (MD -3.08, 95% CI -5.50 to -0.66) and achieving better total effectiveness rate (OR 2.51, 95% CI 1.14 to 5.52) [12].

**Figure 3:**
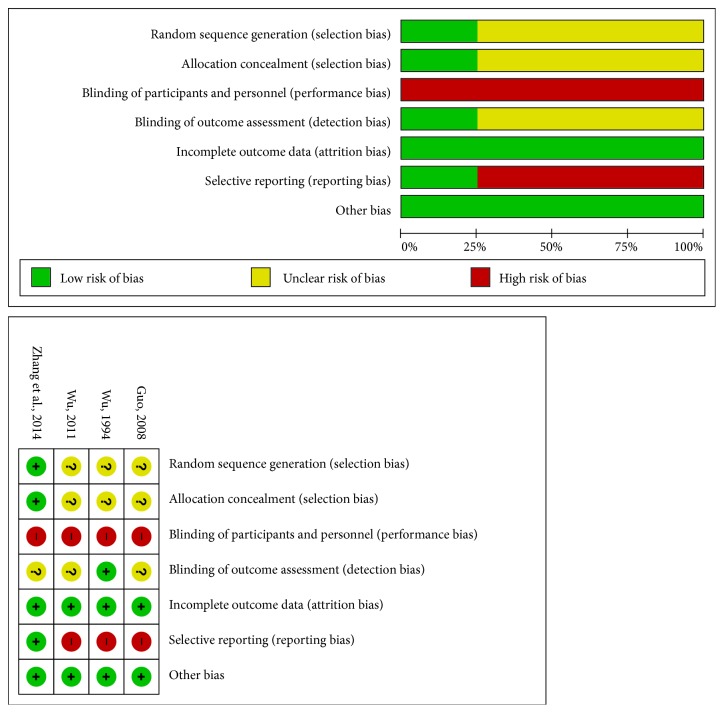
Risk of bias assessment in the four included RCTs.

The risk of bias of the included four RCTs was analyzed in Figure 3. Specifically, it was evaluated based on the published manuscripts since none of the included RCTs registered their protocol with any trial registry. Only one RCT applied proper methods of random sequence generation (random number table) as well as allocation concealment [12]. None of the RCTs blinded participants or personnel. One RCT considered the use of blinding of outcome assessment as it used the laboratory index as an outcome measure [44]. Only one RCT specified dropouts (two in the treatment group and three in the control group) [12]. All RCTs reported similar baseline and none of them reported conflict of interest or funding resources.

The findings from this review on clinical studies indicated that DBKW achieved more benefits in relieving the global symptoms of CP in four weeks, compared to antibiotic drugs [15, 43, 44]. The add-on effects of DBKW to *α*-blocker were also revealed as DBKW could reduce the NIH-CPSI score within six weeks [12]. DBKW is considered safe as only mild adverse events were reported, such as slight fatigue, dizziness and postural hypotension [12]. However, some methodological quality issues of the included RCTs were identified. Only one out of four RCTs applied appropriate randomization and allocation methods [12]. None of the included studies applied placebo as a comparator, therefore, the efficacy of DBKW could not be determined. Since the blinding procedure was not incorporated in the included studies, exaggerated estimation may occur [48]. Various outcome measures were used in the included studies, ranging from total effectiveness rate to laboratory tests, which made data synthesis impossible. As NIH-CPSI is believed to thoroughly reflect the current condition of CP from different aspects (i.e., pain or discomfort, urination, impact of symptoms, and quality of life) [49], it is suggested to use it as a primary outcome measure to monitor intervention's effects on CP patients consistently. In addition, the follow-up procedure is critical in the CP trials because CP is a chronic disease and may undergo frequent recurrence [8]. However, none of the included RCTs involved a follow-up evaluation. Thus, more high quality and properly designed RCTs should be conducted in the future to systematically assess the therapeutic effects of DBKW on the management of CP.

The findings from the current study are consistent with those from another three recently published systematic reviews [50–52]. All the reviews investigated the effects and safety of oral intake of Chinese herbal medicine for CP management. All of them indicated that Chinese herbs could reduce the NIH-CPSI scores when compared to either antibiotic/*α*-blocker only or used as an adjunct therapy to antibiotic/*α*-blocker. They also claimed that oral administration of Chinese herbal medicine was safe for CP patients as only mild adverse events were reported. In addition, similar to our study, all the systematic reviews highlighted the poor quality of included studies and thus recommended rigorously designed RCTs for future studies [50–52].

### 3.2. Multitargeting Actions of DBKW on CP in Experimental Studies

**Figure 4:**
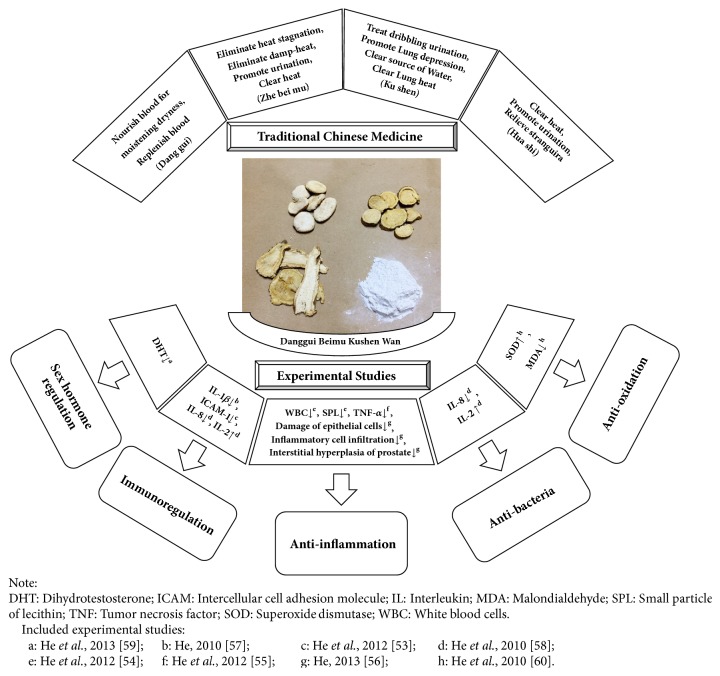
Multitargeting actions of Danggui Beimu Kushen Wan on chronic prostatitis.

Eight included experimental papers were conducted by the same research team and focused on the mechanisms of actions of DBKW for the management of CBP on rats [53–60]. The team set up the CBP model by* Escherichia coli* injection in the prostate of 60 Sprague-Dawley male rats. Basic DBKW decoction was utilized for four weeks from the 15^th^ day of postsurgery whereas norfloxacin was used in the control group. Details of the results are illustrated in Figure 4. The five possible mechanisms of actions of DBKW for CBP concluded in the experimental studies refer to anti-inflammation [54–56], antibacteria [58], antioxidation [60], sex hormone regulation [59], and immunoregulation [53, 57, 58].

DBKW contains more than one herb and therefore it has multiple active compounds [61], such as Angelica sinensis polysaccharides [62], ferulic acid [63], ligustilide [64], matrine [10], oxymatrine [65], sophocarpine [66], and peiminine [67]. Current research stated that combining active compounds would produce additional or even synergistic effects which are superior to a single compound, since concurrent and selective interaction may occur with multiple target proteins of a disease [68, 69]. The recent clinical practice guideline recommended using multimodal therapy (antibiotic with *α*-blocker, nonsteroidal anti-inflammatory drug, or 5*α*-reductase-inhibitors) to manage CP [6, 8]. However, long-term applications of those drugs may lead to adverse events [70–73]. DBKW contains multitargeting agents which could act on more than one pathway of CP at the same time [53–60] (Figure 4). Classic literature supported the assertion that DBKW may have multiactions on the treatment of difficult urination [11]. Compared to the conventional management of CP, DBKW developed less adverse events but better treatment effects that may be due to its multiple natural ingredients with diverse biological activities.

## 4. Conclusion

DBKW has great potential in the treatment of CP evidenced by the current studies described in this review. It is essential to perform high-quality RCTs and network pharmacological studies in future research of DBKW to investigate its clinical effects and mechanisms of actions for CP.

## Figures and Tables

**Table 1:** Characteristics of six included clinical studies.

**Included studies**	**Study type**	**Sample size**	**Dropout**	**Diagnosis**	**Interventions**	**Duration**	**Outcome measurement**	**Adverse events**	**Follow-up**
Guo, 2008 [43]	RCT	T: 85; C: 85	0	CBP, CP/CPPS	T: Modified DBKW decoction, 1 pack, bid; C: Trimethoprim-sulfamethoxazole tablet, 960 mg, bid; levofloxacin, 200 mg, bid	1 month	Total effectiveness rate	NR	No
Wu, 1994 [44]	RCT	T: 60; C: 40	0	CP	T: Modified DBKW decoction, NR; C: Ofloxacin, 200 mg, bid	1 month	EPS-pH; EPS-Lecithin body; EPS-WBC	T: 0; C: 3 (dizziness and nausea)	No
Wu, 2011 [15]	RCT	T: 78; C: 78	0	CBP	T: Modified DBKW decoction, 1 pack, bid; C: Antibiotic drugs, NR	4 weeks	Total effectiveness rate	T: 0; C: 0	No
Zhang *et al.*, 2014 [12]	RCT	T: 60; C: 60	T: 2; C: 3	CP/CPPS	T: Basic DBKW decoction, NR; Terazosin hydrochloride, 2 mg, qn C: Terazosin hydrochloride, 2 mg, qn	6 weeks	NIH-CPSI; Total effectiveness rate	T: 3; C: 4 (Both T and C developed slight fatigue, dizziness and postural hypotension)	No
Li and Yan, 2007 [45]	Case series	45	0	CP/CPPS	Modified DBKW decoction, 1 pack, bid	5 weeks	NIH-CPSI; Total effectiveness rate	NR	No
Wang, 2007 [46]	Case series	120	0	CBP, CP/CPPS	Modified DBKW decoction, 1 pack, bid	1 month	NIH-CPSI; EPS-RT	NR	No

*Note.* C: control group; CP: chronic prostatitis; DBKW: Danggui Beimu Kushen Wan; EPS-RT: expressed prostatic secretions-routine test; EPS-WBC: expressed prostatic secretions test-white blood cells; NIH-CPSI: National Institute of Health of United States-Chronic Prostatitis Symptom Index; NR: not reported; RCT: randomized controlled trial; T: treatment group.

**Table 2:** Details of herb ingredients in the included clinical studies.

**Included studies**	**Herbal ingredients**	
	Basic formula	Individualized
Guo, 2008 [43]	Dang gui 10 g, Zhe bei mu 10 g, Ku shen 15 g, Hua shi 30 g, Wang bu liu xing 10 g	Bai jiang cao 15 g, Pu gong ying 15 g, Xiao hui xiang 10 g, Huo xiang 10 g, Dan shen 15 g, Ba ji tian 15 g, Dang shen 20 g
Wu, 1994 [44]	Dang gui, Zhe bei mu, Ku shen, Huang bai, Pu gong ying, Shi chang pu, Mu dan pi, Shui zhi, Wu yao (no dosage provided)	Yi yi ren, Fu zi, Bai jiang cao, Gui zhi, Fu ling, Chi shao, Tao ren (no dosage provided)
Wu, 2011 [15]	Dang gui 15 g, Zhe bei mu 15 g, Ku shen 10 g, Hua shi 10 g, Chi shao 12 g, Hong hua 6 g, Huang bai 10 g, Chuan niu xi 10 g, Wang bu liu xing 6 g	N/A
Zhang *et al.*, 2014 [12]	Dang gui 24 g, Zhe bei mu 24 g, Ku shen 24 g, Hua shi 3 g	N/A
Li and Yan, 2007 [45]	Dang gui 10 g, Zhe bei mu 10 g, Ku shen 10 g, Hua shi 15 g	Chuan niu xi, Tao ren, Hong Hua, Nu zhen zi, He shou wu, Huang jing (no dosage provided)
Wang, 2007 [46]	Dang gui 15 g, Zhe bei mu 15 g, Ku shen 15 g, Hua shi 12 g, Pu huang 10 g, Dan shen 15 g, Pu gong ying 15 g, Chuan niu xi 15 g, Chuan lian zi 10 g, Chuan shan jia 6 g, Li zhi he 12 g	Gou qi zi, Huang jing, Yin yang huo, Ba ji tian (no dosage provided)

*Note.* N/A: not applied; herb ingredients are listed as Chinese pinyin. Corresponding Latin names refer to the nomenclature list of commonly used Chinese herbal medicines published by the Chinese Medicine Board of Australia [47].
